# A bone tumor‐like chest wall mass lesion with pathological rib fractures observed 13 years after lung stereotactic body radiotherapy: A case report

**DOI:** 10.1111/1759-7714.15419

**Published:** 2024-08-07

**Authors:** Masaki Matsuda, Jiro Ichikawa, Takafumi Komiyama, Kojiro Onohara, Masahide Saito, Hikaru Nemoto, Mizuki Kubota, Hiroshi Onishi

**Affiliations:** ^1^ Department of Radiology University of Yamanashi Yamanashi Japan; ^2^ Department of Orthopaedic Surgery University of Yamanashi Yamanashi Japan; ^3^ Department of Pathology University of Yamanashi Yamanashi Japan

**Keywords:** chest wall toxicities, non‐small cell lung cancer, radiation‐induced sarcoma, rib fracture, stereotactic body radiotherapy

## Abstract

Although stereotactic body radiotherapy (SBRT) is a curative treatment option for stage I non‐small cell lung cancer (NSCLC), limited data are available regarding chest wall (CW) toxicities during an extended follow‐up of over 10 years. We report an unusual case of a bone tumor‐like CW mass lesion with pathological rib fractures observed 13 years after SBRT for peripheral lung cancer. Despite the initial suspicion of radiation‐induced sarcoma, a subsequent incisional biopsy revealed no evidence of malignancy, and a definitive diagnosis of osteonecrosis was made. Thus, long‐term observation of over 10 years is required to identify late chronic complications following SBRT.

## Graphical abstract

Here, we present an unusual case of a bone tumor‐like chest wall mass lesion with pathological rib fractures observed 13 years after lung stereotactic body radiotherapy (SBRT) for peripheral lung cancer, masquerading as radiation‐induced sarcoma. An incisional biopsy revealed no evidence of malignancy with a definitive diagnosis of osteonecrosis. Long‐term observation of over 10 years is required to identify late chronic complications following SBRT.

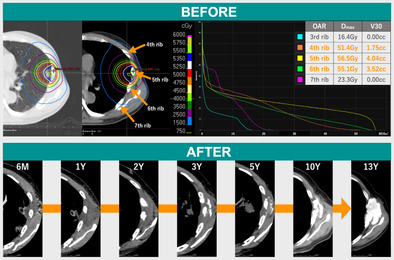

## INTRODUCTION

Stereotactic body radiotherapy (SBRT) is a curative and minimally invasive treatment option for stage I non‐small cell lung cancer (NSCLC), with excellent local control rates, even over long‐term follow‐up exceeding 5 years.^1^, ^2^ However, in the long‐term setting, it is associated with various complications depending on the irradiated site. Chest wall (CW) toxicities, including CW pain and rib fractures, are common adverse events after lung SBRT, while several studies have shown that tumorous extrapulmonary fibrosis can occur in the CW.^3^, ^4^ Currently, data regarding CW toxicities occurring over an extended follow‐up of over 10 years are limited. Here, we report a case of a bone tumor‐like CW mass lesion with pathological rib fractures occurring a long time after lung SBRT.

## CASE REPORT

A 64‐year‐old Japanese man with a history of hypertension and chronic hepatitis C underwent computed tomography (CT) in August 2007 that revealed a peripheral 15‐mm ground‐glass nodule in the left upper lobe. Follow‐up CT revealed a solid component within the nodule. Fluorodeoxyglucose‐positron emission tomography (FDG‐PET)/CT revealed mild FDG uptake and transbronchial lung biopsy revealed a bronchioloalveolar carcinoma. The patient was diagnosed with T1N0M0 carcinoma according to the TNM classification of the Union for International Cancer Control, sixth edition.

**FIGURE 1:**
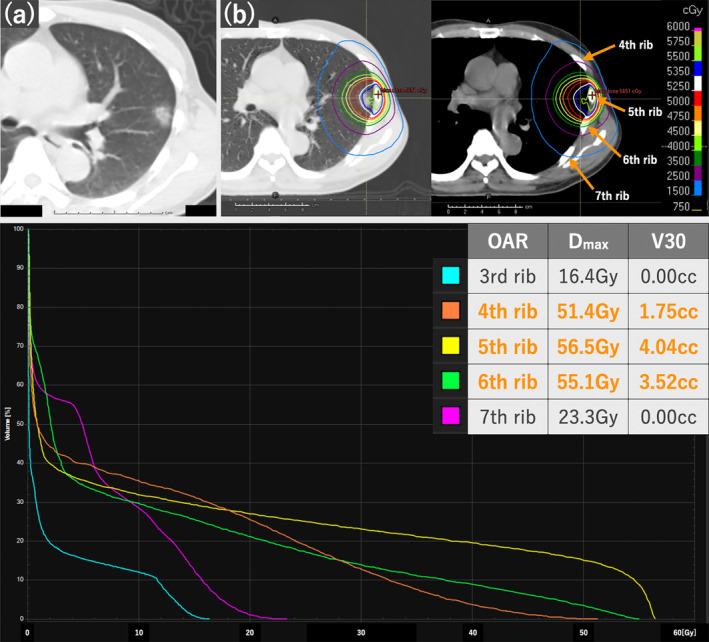
(a) Chest computed tomography (CT) scan showing a tumor in the left upper lobe adjacent to the chest wall. (b) Stereotactic body radiotherapy (SBRT) was performed with 48 Gy in four fractions; isodose lines of 100% (red), 50% (purple), and 30% (cobalt) are shown. Dose‐volume histogram of each rib; third rib (light blue), fourth rib (orange), fifth rib (yellow), sixth rib (light green), and seventh rib (pink).

The patient had a 5‐pack‐year smoking history. Physical examination results were unremarkable. As the patient declined surgery, he was referred for radical radiotherapy and was administered SBRT comprising three‐dimensional conformal radiotherapy with a linear accelerator in March 2008 (Figure 1). A total dose of 48 Gy in four fractions was prescribed to cover >95% of the internal target volume. The maximum doses (D_max_) to the CW and ribs were 56.5 Gy each, and the corresponding volumes receiving 30 Gy (V30) in these structures were 46.1 and 9.26 cc, respectively. No specific dose constraints were applied to the CW or ribs.

**FIGURE 2:**
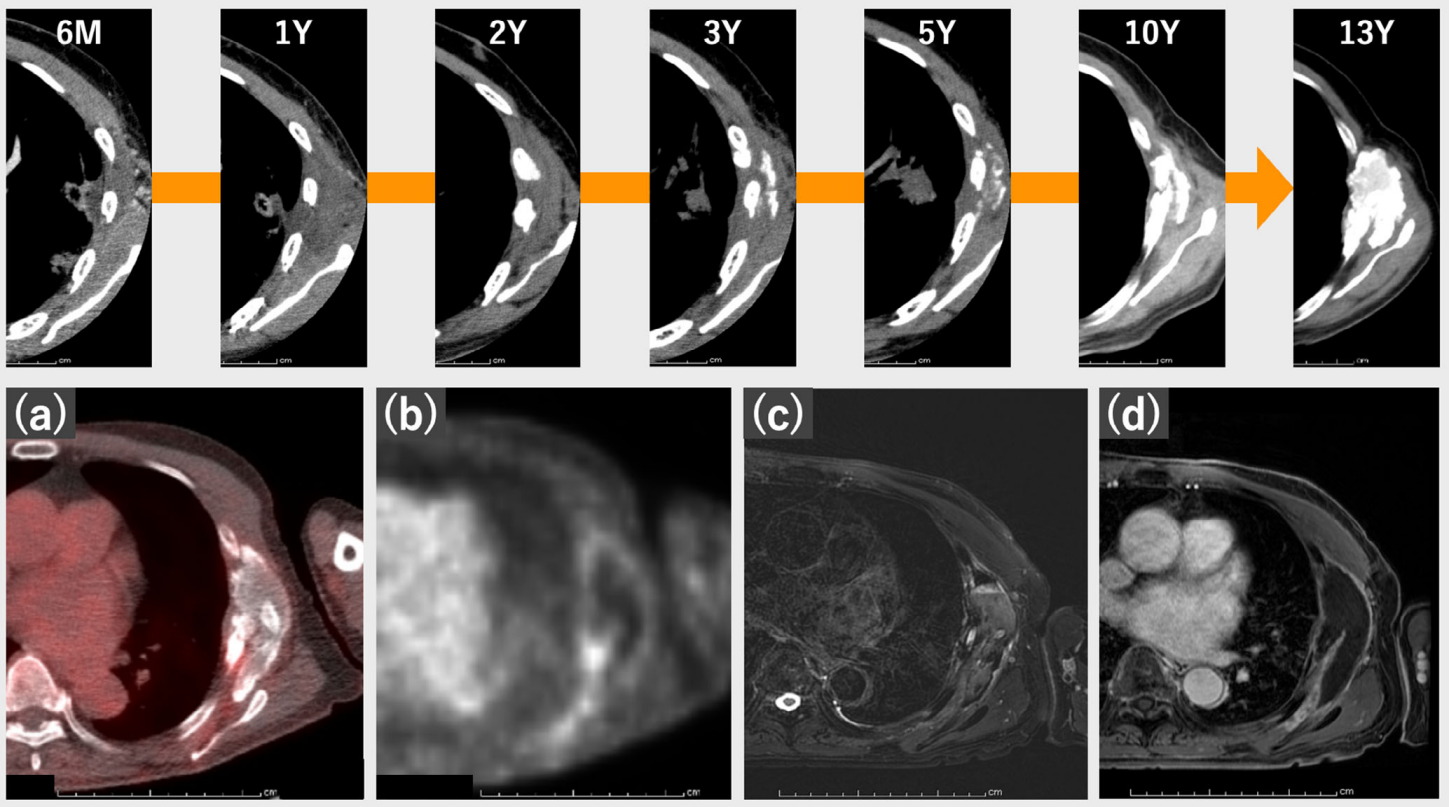
The time course of the chest wall on computed tomography (CT) imaging during the 13 years following stereotactic body radiotherapy (SBRT), showing a tumor‐like mass which increased in size and diffuse calcification. (a, b) Fluorodeoxyglucose‐positron emission tomography‐computed tomography (FDG‐PET/CT) showing low FDG uptake. (c) Fat‐saturated T2‐weighted images showing low signal intensity with fluid–fluid levels. (d) Fat‐saturated contrast‐enhanced T1‐weighted images showing no enhancement.

Four months post‐SBRT, the patient experienced CW pain, requiring analgesia with oral COX inhibitors (loxoprofen) for several years. Follow‐up CT revealed CW edema appearing 6 months post‐SBRT and rib fractures near the tumor occurring 1–3 years post‐SBRT, with subsequent soft‐tissue hyperplasia and heterotopic calcification within 10 years, followed by CW mass progression 13 years post‐SBRT. Figure 2 illustrates the time course of CT. The tumor‐like mass exhibited diffuse calcification and low FDG uptake. Magnetic resonance imaging (MRI) revealed low signal intensity with fluid–fluid levels on fat‐saturated T2‐weighted images without enhancement.

**FIGURE 3:**
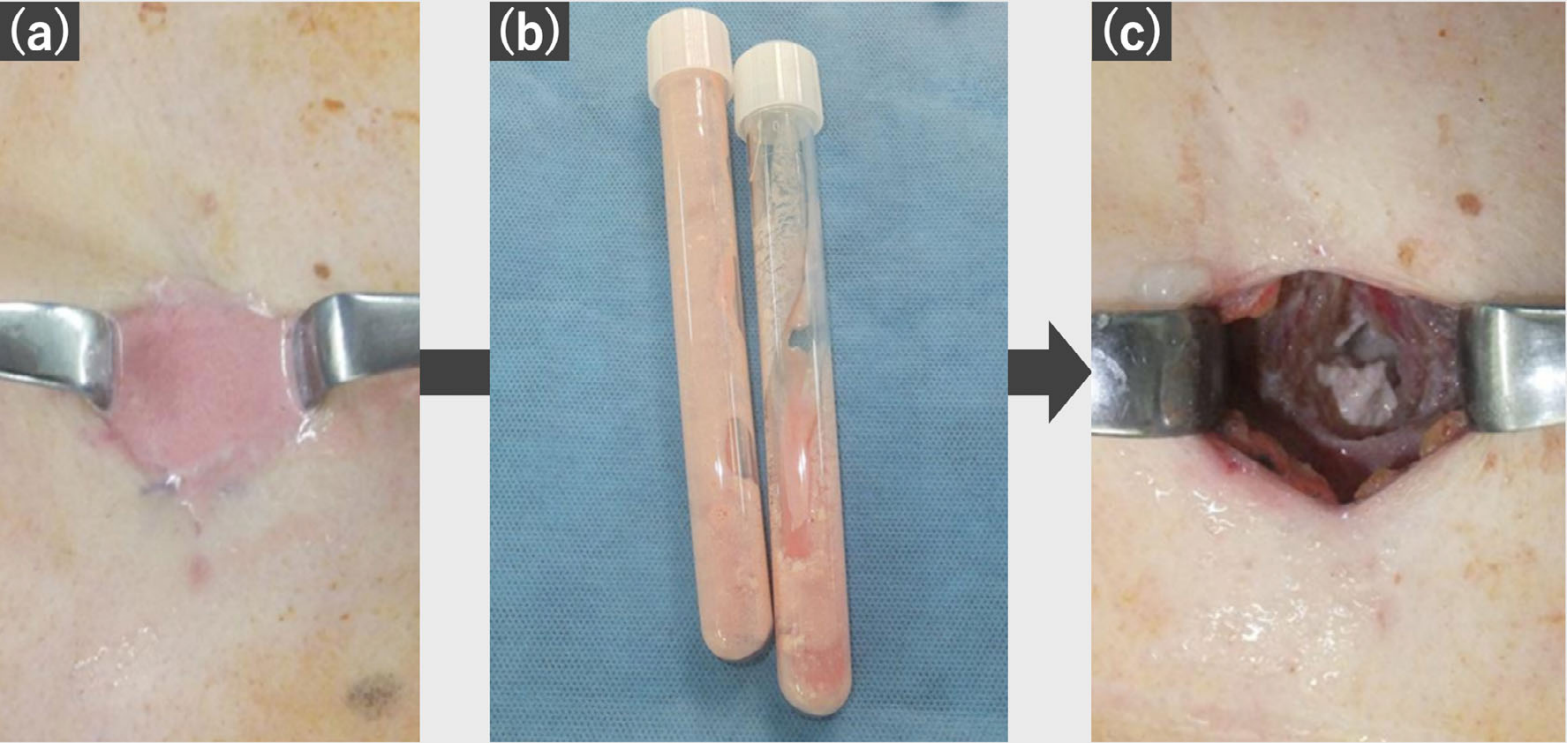
(a, b) Light‐pink cloudy fluid was obtained from an incision in the chest wall; subsequent cytology revealed class I features.

**FIGURE 4:**
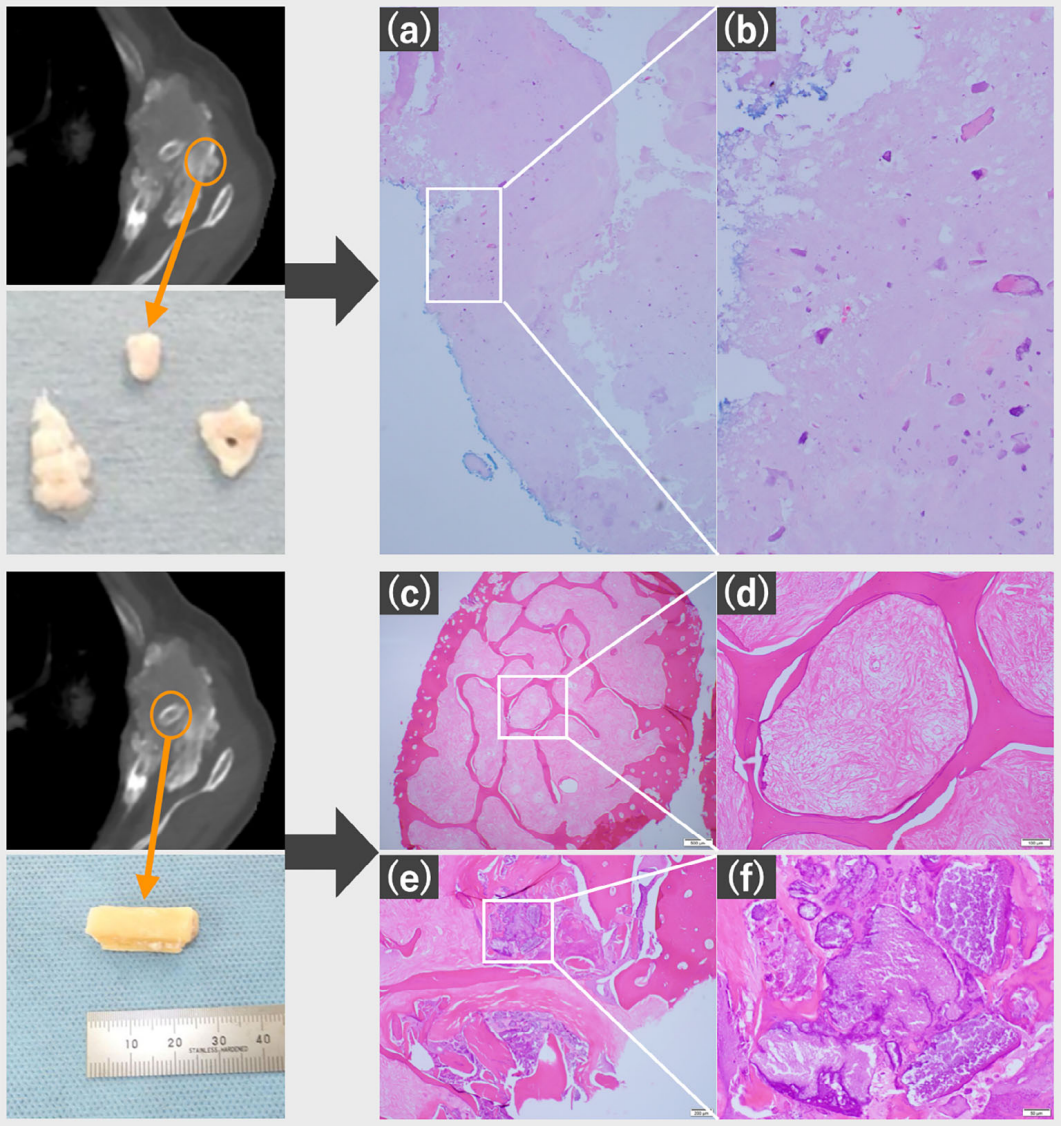
Pathological findings of incisional biopsy. (a, b) The mass specimen comprised granular calcification inside the degenerated cartilage matrix‐like tissue. (c, d) Isolated rib fragment included hyalinized fibrosis inside the trabecular bone structure (e, f) and coarse calcification.

Incisional biopsy performed to exclude secondary malignancies revealed a light‐pink cloudy fluid whose cytology revealed class I features (Figure 3). Biopsy specimens showed granular calcification within degenerated cartilage matrix‐like tissue. The isolated rib fragments exhibited hyalinized fibrosis and coarse calcification within the trabecular bone structure (Figure 4). Since the mass showed no significant changes on 1‐year post‐biopsy CT, additional biopsy was deemed unnecessary.

## DISCUSSION

SBRT is an effective treatment for stage I NSCLC, allowing delivery of ablative radiation doses to the tumor. Although the long‐term effectiveness of SBRT is being actively studied, its long‐term complications remain to be elucidated. SBRT has the potential to cause CW toxicity, particularly when used to treat peripheral lung cancer near the CW. A systematic review estimated the pooled incidences of CW pain and rib fractures to be 8.94% and 5.27%, with median times‐to‐onset ranging from 3–13 months and 3–34.8 months, respectively.^5^ One study revealed the emergence of soft‐tissue masses in 2.4% of patients with pulmonary tumors near the CW within 3–36 months post‐SBRT. Percutaneous needle biopsy was necessary for three patients to exclude tumor recurrence or secondary malignancy, all of which revealed benign soft‐tissue fibrotic changes.^3^ Another study reported that 67% of rib fractures developed surrounding tumorous soft‐tissue fibrosis, 62% of whom developed heterotopic ossification during the healing process.^4^ However, both studies had a follow‐up time of <10 years. Conversely, our study reports a CW mass that enlarged after a decade.

We considered secondary sarcoma a differential diagnosis. Radiation‐induced sarcoma is rare, accounting for 0.03%–0.9% of all irradiated patients within 15 years after radiotherapy.^6^, ^7^ The most common histological subtype is radiation‐induced osteosarcoma, which typically develops at a dose of >30 Gy;^8^ however, no data exists regarding radiation‐induced sarcoma after SBRT. The reported imaging features of radiation‐induced osteosarcoma include (a) mixed lytic and sclerotic lesions on CT; (b) aggressive periosteal reactions characterized by spiculated, disorganized, laminated, or Codman triangle patterns on CT; (c) increased FDG uptake on FDG‐PET/CT; and (d) heterogeneous signal intensity on T1‐ and T2‐weighted MRI with contrast enhancement.^9^ Although our imaging findings were not consistent with these features, mass progression within a previously irradiated site rendered the exclusion of radiation‐induced sarcoma challenging. Additionally, pathological examination revealed no evidence of malignancy and confirmed the definitive diagnosis of osteonecrosis. The growing tumor‐like lesion caused by osteonecrosis can be result of a complex interplay between chronic inflammation and reparative processes, leading to the proliferation of fibroblasts and other reparative cells.

The dose constraints for the CW and ribs remain unclear. According to the VALOR study, CW dose‐volume limits are D_max_ of 56.7–60.4 Gy and V30 < 30 cc, with a D_max_ of 50–57 Gy for the ribs.^10^ Meanwhile, the NCCN guidelines recommend a maximum rib dose of 40 Gy in 4 fractions, based on requirements from RTOG trials.^11^ Although tumor coverage should not be compromised for CW sparing, dose restriction is recommended to minimize CW toxicities.^12^ Thus, we should have given more consideration to reducing CW dose, such as using intensity‐modulated radiotherapy.

In conclusion, this is the first case report to demonstrate a bone tumor‐like CW mass lesion with pathological rib fractures 13 years after lung SBRT. This experience suggests that long‐term observation of over 10 years is required to identify late chronic complications following SBRT, including radiation‐induced sarcoma. Additionally, biopsy should be considered for an accurate diagnosis.

## AUTHOR CONTRIBUTIONS

Conceptualization: Masaki Matsuda. Supervision: Jiro Ichikawa and Hiroshi Onishi. Writing—original draft: Masaki Matsuda. Writing—review and editing: Jiro Ichikawa, Takafumi Komiyama and Hiroshi Onishi. Resources: Kojiro Onohara and Mizuki Kubota. Data curation: Masahide Saito and Hikaru Nemoto. All authors revised the report, commented on drafts of the manuscript, and approved the final report to be published.

## CONFLICT OF INTEREST STATEMENT

The authors declare that this study was conducted without any commercial or financial relationships that could be interpreted as conflicts of interest.

## Data Availability

The data that support the findings of this study are available from the corresponding author, Masaki Matsuda, upon reasonable request.
